# A U.S. isolate of *Theileria orientalis*, Ikeda genotype, is transmitted to cattle by the invasive Asian longhorned tick, *Haemaphysalis longicornis*

**DOI:** 10.1186/s13071-021-04659-9

**Published:** 2021-03-16

**Authors:** Kelcey D. Dinkel, David R. Herndon, Susan M. Noh, Kevin K. Lahmers, S. Michelle Todd, Massaro W. Ueti, Glen A. Scoles, Kathleen L. Mason, Lindsay M. Fry

**Affiliations:** 1 Department of Veterinary Microbiology & Pathology, Washington State University, Pullman, WA USA; 2 United States, Department of Agriculture, Agricultural Research Service, Animal Disease Research Unit, Pullman, WA USA; 3 Department of Biomedical Sciences and Pathobiology, Virginia–Maryland College of Veterinary Medicine, Blacksburg, VA USA; 4 Present Address: United States Department of Agriculture, Agricultural Research Service, Invasive Insect Biocontrol and Behavior Laboratory, Beltsville, MD USA

**Keywords:** *Theileria orientalis*, Ikeda genotype, *Haemaphysalis longicornis*, Asian longhorned tick, Transmission, Cattle

## Abstract

**Background:**

*Theileria orientalis* is a tick-borne hemoparasite that causes anemia, ill thrift, and death in cattle globally. The Ikeda strain of *T.* *orientalis* is more virulent than other strains, leading to severe clinical signs and death of up to 5% of affected animals. Within the Asia–Pacific region, where it affects 25% of Australian cattle, *T.* *orientalis* Ikeda has a significant economic impact on the cattle industry. In 2017, *T.* *orientalis* Ikeda was detected in a cattle herd in Albermarle County, Virginia, United States. Months earlier, the U.S. was alerted to the invasion of the Asian longhorned tick, *Haemaphysalis longicornis,* throughout the eastern U.S. Abundant *H.* *longicornis* ticks were identified on cattle in the *T.* *orientalis-*affected herd in VA, and a subset of ticks from the environment were PCR-positive for *T.* *orientalis* Ikeda. A strain of *T.* *orientalis* from a previous U.S. outbreak was not transmissible by *H.* *longicornis*; however, *H.* *longicornis* is the primary tick vector of *T.* *orientalis* Ikeda in other regions of the world. Thus, the objective of this study was to determine whether invasive *H.* *longicornis* ticks in the U.S. are competent vectors of *T.* *orientalis* Ikeda.

**Methods:**

Nymphal *H.* *longicornis* ticks were fed on a splenectomized calf infected with the VA-U.S.-*T.* *orientalis* Ikeda strain. After molting, a subset of adult ticks from this cohort were dissected, and salivary glands assayed for *T.* *orientalis* Ikeda via qPCR. The remaining adult ticks from the group were allowed to feed on three calves. Calves were subsequently monitored for *T.* *orientalis* Ikeda infection via blood smear cytology and PCR.

**Results:**

After acquisition feeding on a VA-U.S.-*T.* *orientalis* Ikeda-infected calf as nymphs, a subset of molted adult tick salivary glands tested positive by qPCR for *T.* *orientalis* Ikeda. Adult ticks from the same cohort successfully transmitted *T.* *orientalis* Ikeda to 3/3 naïve calves, each of which developed parasitemia reaching 0.4–0.9%.

**Conclusions:**

Our findings demonstrate that U.S. *H.* *longicornis* ticks are competent vectors of the VA-U.S.-*T.* *orientalis* Ikeda strain. This data provides important information for the U.S. cattle industry regarding the potential spread of this parasite and the necessity of enhanced surveillance and control measures.
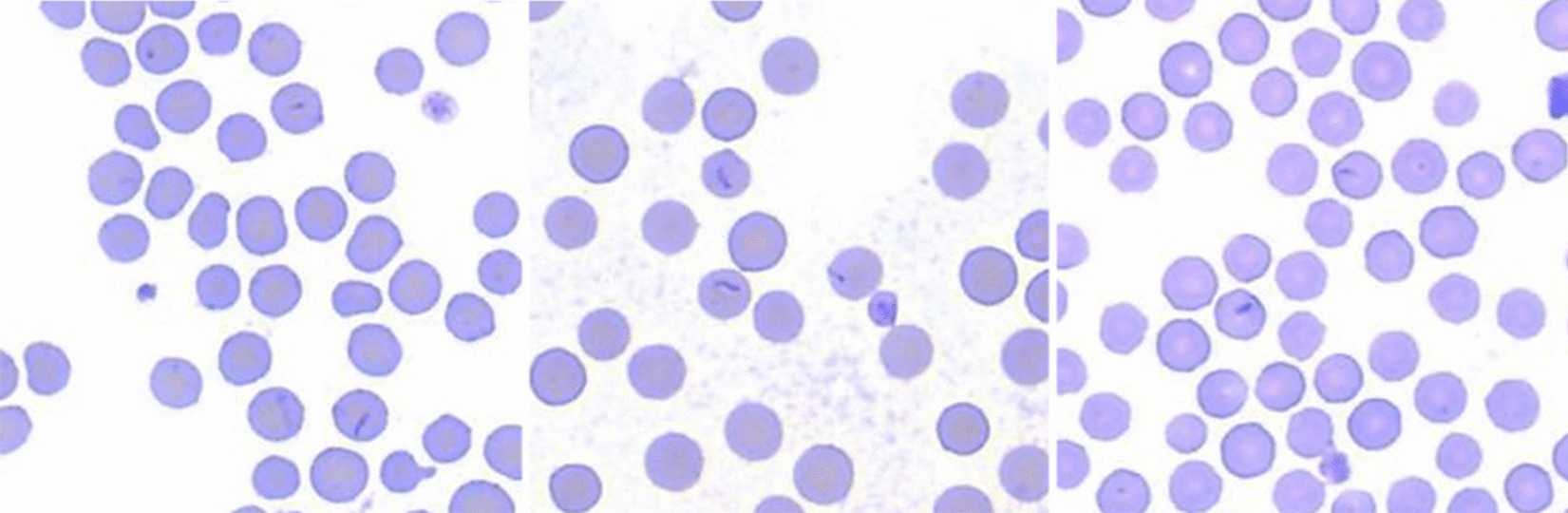

**Supplementary Information:**

The online version contains supplementary material available at 10.1186/s13071-021-04659-9.

## Background

*Theileria orientalis* comprises a diverse group of non-transforming, bovine hemoparasites that were formerly classified as *T.* *sergenti* and *T.* *buffeli* [1]. Phylogenetic analysis using major piroplasm surface protein (MPSP) sequences of numerous isolates identified 11 allelic genotypes [2], with the Buffeli, Chitose, p32, and Ikeda genotypes being most prevalent in Japan, Australia, New Zealand, and the U.S [2, 3]. *T.* *orientalis* genotypes vary widely in virulence [4]. The Ikeda genotype results in severe infection in 1–6% of infected animals [5, 6], while infection with the Buffeli genotype is almost always clinically silent [3, 5]. Moderate to severe infections are characterized by erythrocyte destruction, leading to anemia and hypoxia. Severely affected animals often exhibit pyrexia, weakness, pallor, and increased heart and respiratory rates [7, 8]. Abortion is a common sequela to *T.* *orientalis* Ikeda infection, although transplacental transmission of the parasite from cows to calves is only observed in 10% of cases [9, 10].

The primary tick vector of *T.* *orientalis* is *Haemaphysalis longicornis* (AKA the Asian longhorned tick) [3, 11]; however, other tick species [3, 12] and mechanical vectors, including contaminated needles [13], biting flies [13], and lice [14], have been implicated in parasite spread. Regardless, *T.* *orientalis* Ikeda can spread rapidly and cause significant losses when introduced to naïve cattle in areas with competent tick vectors. *T.* *orientalis* Ikeda was first identified in Australia in 2011 [15], and by 2014, outbreaks of the disease had affected approximately 25% of Australian cattle [16, 17]. It is estimated that the indirect costs of *T.* *orientalis* Ikeda to the Australian beef industry, comprised of reduced meat and milk yields [16, 18], is $19.6 million per year [19]. A similar, sudden emergence of the *T.* *orientalis* Ikeda genotype occurred in New Zealand beginning in 2012 [20], with periparturient and lactating dairy cows and young calves exhibiting the highest morbidity and mortality [20]. *T.* *orientalis* Ikeda is now the most important cause of anemia in New Zealand cattle [7].

In the United States, *T.* *orientalis* outbreaks have been historically rare, and, until 2017, were attributed to strains closely related to the clinically benign Buffeli genotype [1, 21, 22]. In 2017, a cow-calf beef herd in Albemarle County, VA experienced an outbreak of febrile illness. Six animals died, and examination of a 7th clinically ill animal revealed anemia and lethargy. Blood from this animal was positive for *T.* *orientalis*. The Ikeda genotype was identified from the clinically affected animal and herd mates [23]. In parallel, ticks collected from a calf on the index farm were identified as *H.* *longicornis*, the first report of this tick outside of New Jersey*.*

*H. longicornis* is native to countries within East Asia, including Russia, China, Korea, and Japan [24]. As *H.* *longicornis* can survive within a diverse climactic range, and many populations are capable of parthenogenetic reproduction, it has successfully invaded and become established in many countries within the Asia–Pacific region, including Australia and New Zealand [24]. Recently, *H.* *longicornis* invasion of the United States has been confirmed [25]. In addition to its presence on the *T.* *orientalis* Ikeda outbreak index farm in Virginia, it has been identified in New Jersey [25], New York [26, 27], Pennsylvania [28], Maryland [28], Delaware [29], Connecticut [28], North Carolina [30], West Virginia [31], Tennessee [29], Kentucky [29], Arkansas [31], Rhode Island [29], Ohio [29], and South Carolina [29], and is believed to have been present within the eastern United States for at least 8 years, having initially been misclassified as *H.* *leporispalustris* [31]*.*

In addition to *H.* *longicornis,* several other tick species are competent biological vectors of *T.* *orientalis*, including other species of *Haemaphysalis* ticks [3] and *Rhipicephalus (Boophilus) microplus* [12]*.* Interestingly, *T.* *orientalis* Buffeli isolated from a historical U.S. outbreak was not transmissible by Korean *H.* *longicornis* ticks [1, 32]. Although *H.* *longicornis* ticks from VA were PCR-positive for *T.* *orientalis* Ikeda [33], it is not yet known whether these ticks are the source of *T.* *orientalis* transmission to cattle in VA. If *H.* *longicornis* proves to be a competent vector of *T.* *orientalis* Ikeda in the U.S., increased tick control practices and vigilance regarding cattle health may be required to prevent disease spread and losses similar to those experienced by cattle producers in Australia and New Zealand. Thus, the objective of this study was to determine whether the strain of *H.* *longicornis* ticks that has spread throughout the U.S. can acquire and transmit the *T.* *orientalis* Ikeda-VA isolate. The results of this study will provide important information for U.S. cattle producers regarding the potential threat of pathogen transmission to cattle by the invasive Asian longhorned tick.

## Materials and methods

### Cattle

Four, 2–3 month-old Holstein–Friesian steer calves were utilized in this study. Pre-infection complete blood counts (CBCs) and serum chemistry panels were normal, and all calves tested negative on a pre-infection *Anaplasma marginale*/*Anaplasma centrale* competitive enzyme linked immunosorbent assay (cELISA) test (VMRD, Pullman, WA). PCR for the *T.* *orientalis* Ikeda major piroplasm surface protein (MPSP) (described below) performed on pre-infection peripheral blood samples from each calf was negative. The experimental protocol was approved by the University of Idaho Institutional Animal Care and Use Committee, Protocol number 2018-46.

One steer, #1697, underwent splenectomy at the Washington State University Veterinary Teaching Hospital, and, to ensure full recovery, was placed on stall rest for 4 weeks before proceeding with the experiment. The splenectomy was performed under the supervision of a board-certified agricultural animal internist using standard surgical, anesthetic, and analgesic protocols. Once recovered, the calf was infected intravenously with 6 mL of *T.* *orientalis* Ikeda blood stabilate (Abermarle, VA 2018). Beginning 10 days after infection, rectal temperature, pulse, respiratory rate, lymph node size, and mucous membrane color were assessed and recorded daily, and a CBC, chemistry panel, and *T.* *orientalis* peripheral blood PCR and qPCR assays (described below) were performed weekly. Diff-Quik-stained blood smears and packed cell volume were assessed 2–3 times per week until the calf became positive by PCR, and were measured daily thereafter. Monitoring of steers and *T.* *orientalis* infection kinetics were similarly performed for tick acquisition and transmission feeds. Following tick removal and confirmation of infection, the calf was euthanized via intravenous administration of sodium pentobarbital (Fatal Plus®, Vortech Pharmaceuticals, Michigan, USA).

### *T. orientalis* blood stabilate

Whole blood from a *T.* *orientalis* Ikeda PCR-positive calf identified during the VA outbreak was collected into citrate phosphate dextrose adenine (CPDA) anticoagulant via jugular venipuncture. Following collection, blood was centrifuged at 1800×*g* for 10 min, and the plasma and buffy coat removed. The remaining erythrocyte fraction was washed three times in filter-sterilized (0.22 µm) Puck’s saline G (pH 7.2). Finally, 20% polyvinylpyrrolidone-40 (Sigma, St. Louis, MO, USA) was added to the washed erythrocytes and 1.5 mL aliquots of the 1:1 blood/PVP mixture were transferred to 2 mL cryovials. The mixture was cryopreserved at – 80 °C for later use, and stabilates were thawed rapidly immediately prior to inoculation.

### *H. longicornis* colony maintenance

*H. longicornis* adult and nymphal ticks were collected from the field in New Jersey by Dr. Dana Price (Rutgers University, New Jersey) and kindly provided to the USDA-ARS Animal Disease Research Unit in Pullman, WA for colony rearing. To rear all life stages, ticks were fed on Holstein-Friesian steer calves under a secure cloth or stockinette patch attached by adhesive. Fed ticks were collected and held in an incubator at 26 °C to allow molting and tanning. Colony ticks were held for long-term storage at 15 °C and experimental ticks were maintained at 26 °C until used for acquisition and transmission feeds. The ticks used in these experiments were reared from eggs in our facility in Moscow, ID. The colony, which is composed entirely of females and reproduces parthenogenetically, had been reared for one generation (F1) as of the date of this transmission trial.

### *H. longicornis* acquisition feed

For acquisition feeding, nymphs were applied to splenectomized steer #1697 in two batches. Prior to feeding, an 8.5″ × 8.0″ patch divided into two equal sections was applied to the back of the calf. Batch #1 was applied 13 days after first detection of *T.* *orientalis* by peripheral blood PCR (see below). Batch #2 was applied 6 days later after visualization of piroplasms within erythrocytes on Diff-Quik-stained blood smears. For each batch, 154 nymphs were applied to one section of the cloth patch. Nymphs fed to repletion over a 4–8 day period. During this time, engorged nymphs were collected and held as described above to allow molting into adults.

### *H. longicornis* transmission feed

Following acquisition feeding, nymphs were held for approximately 1 month to allow molting. Post-molt, adult ticks from both batches were divided evenly into three groups and allowed to feed on three, spleen-intact steers (#1718, #1726, and #1727). Prior to tick application, an 8.5″ × 8.0″ patch was applied to the right and left flanks of each calf using adhesive. For transmission feeding, 42 ticks from Batch #1 and 28 ticks from Batch #2 were applied to each calf in the front left and right patches, respectively.

To confirm *T.* *orientalis* acquisition and maturation, a subset of adult ticks from each batch were allowed to feed for 4 days to stimulate salivary gland development and parasite replication, and were then collected and dissected to harvest salivary glands. Salivary glands were placed into individual 1.5 mL microfuge tubes on ice containing Phosphate Buffered Saline (PBS; Gibco) and stored at – 20 °C until analysis by qPCR as described below.

### *T. orientalis* MPSP PCR

Frozen samples were extracted using the QIAamp DNA mini kit (Qiagen, Hilden, Germany) following the blood or body fluids spin protocol (blood and salivary glands) or the tissue protocol (larvae) using proteinase K. EDTA anticoagulated blood (100 µL) and salivary gland pairs dissected from acquisition fed adults were brought up to 200 µL with PBS. Elution was performed with 50 µL of pre-warmed buffer AE and 2 min incubation prior to centrifugation; this step was repeated for blood samples. Detection of the MPSP gene by conventional PCR was carried out in reactions composed of 22.5 µL of AccuPrime™ *Pfx* SuperMix (Thermo Fisher Scientific, Waltham, MA, USA), 1.5 µL of 10 uM each MPSP primer [23] and 2 µL of DNA template. Reaction conditions were as follows: 5 min at 95 °C followed by 35 cycles of 15 s at 95 °C, 5 s at 57 °C and 1 min at 68 °C. Ten microliters of the final reaction volume was electrophoresed on a 1.25% agarose gel. Positive controls (blood stabilate) and positive samples produced fragments of the expected size of 776 bp and negative controls showed no signs of amplification (Additional file 1: Figure S1). Positive samples (one date per animal and blood stabilate control) were verified as *T.* *orientalis* with bidirectional Sanger sequencing using PCR primers (Eurofins Genomics, Louiseville KY). Sequences were trimmed and merged for consensus to create consensus sequences (Sequencher, GeneCodes, Ann Arbor, MI, USA).

### *T. orientalis* qPCR

Real-time PCR targeting the *T.* *orientalis* MPSP gene was completed on DNA extracted from blood of the four steers and from salivary glands of ticks fed on the three transmission-fed steers. PCR reaction components consisted of TaqMan™ Environmental Master Mix 2.0 (Thermo Fisher Scientific), 300 µM of Forward Primer [34] (5′-GCA AAC AAG GAT TTG CAC GC-3′), 300 µM of Reverse Primer (5′-TGT GAG ACT CAA TGC GCC TAG A-3′), 100 µM of Probe (5′-NED–TCG ACA AGT TCT CAC CAC-MGB-NFQ-3′), and 2 µL of DNA in a 20 µL reaction. Serial ten-fold dilutions of plasmid DNA pASK-IBA2 (IBA Life Sciences, Goettingen, Germany) ligated with a partial *T.* *orientalis* Ikeda MPSP gene were included in the assay as a standard curve. qPCR was run on a 7500 Fast Real-Time PCR System (Thermo Fisher Scientific) using standard mode and the following cycling conditions: 10 min at 95C followed by 45 cycles of 15 s at 95C and 1 min at 60C (Additional file 2: table S1).

## Results

### Infection of a splenectomized calf with *T. orientalis* Ikeda blood stabilate results in microscopically detectable parasites and anemia

**Fig. 1:**
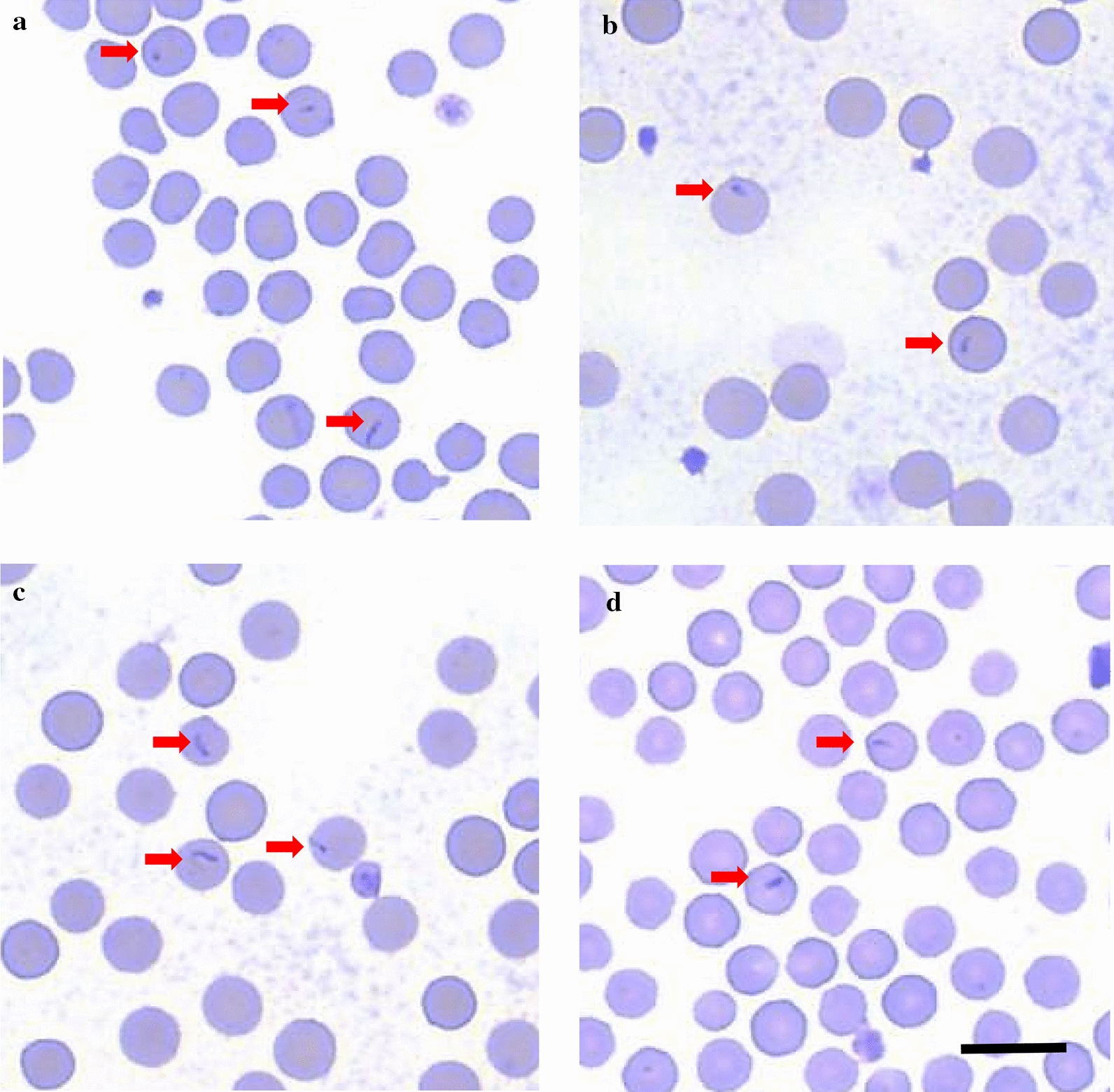
Representative blood smears from calves 1697, 1718, 1726, and 1727 following *T. orientalis* Ikeda-infection**.** In each calf, few erythrocytes contain 1–2.5 µm × 0.5 µm, tear-drop shaped, intracellular piroplasms (arrows). No other evidence of anemia, erythrocyte destruction, or cellular regeneration is present. **a** calf 1697, **b** calf 1718, **c** calf 1726, **d** calf 1727. Scale bar: 10 µm

**Fig. 2:**
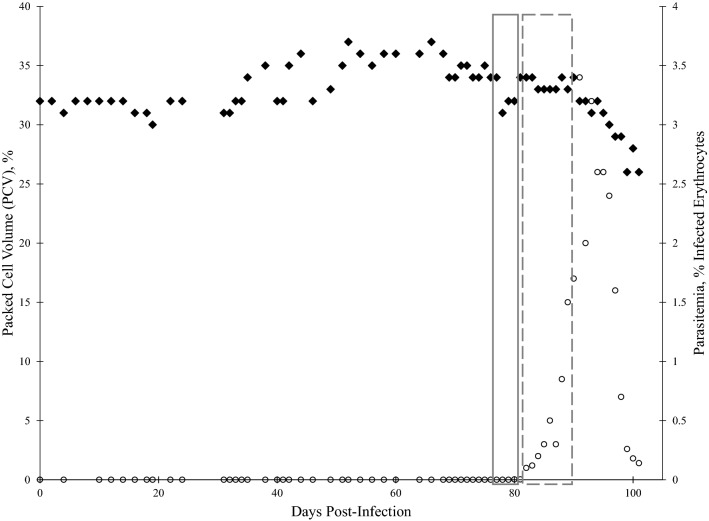
Packed cell volume (PCV) and peripheral blood percent parasitemia of erythrocytes (PPE) of calf 1697 following *T. orientalis* Ikeda blood stabilate inoculation**.** PCV and Diff-quick-stained blood smears were evaluated following intravenous inoculation with *T. orientalis-*Ikeda-infected blood stabilate. PCV (♦ left y-axis) and PPE (○ right y-axis) are depicted as percentages, and each value is derived from analysis of a single blood sample taken on the indicated day post-inoculation. Grey boxes indicate timeline of nymphal tick acquisition feeds. Solid box: Batch #1, Dashed box: Batch #2

**Fig. 3:**
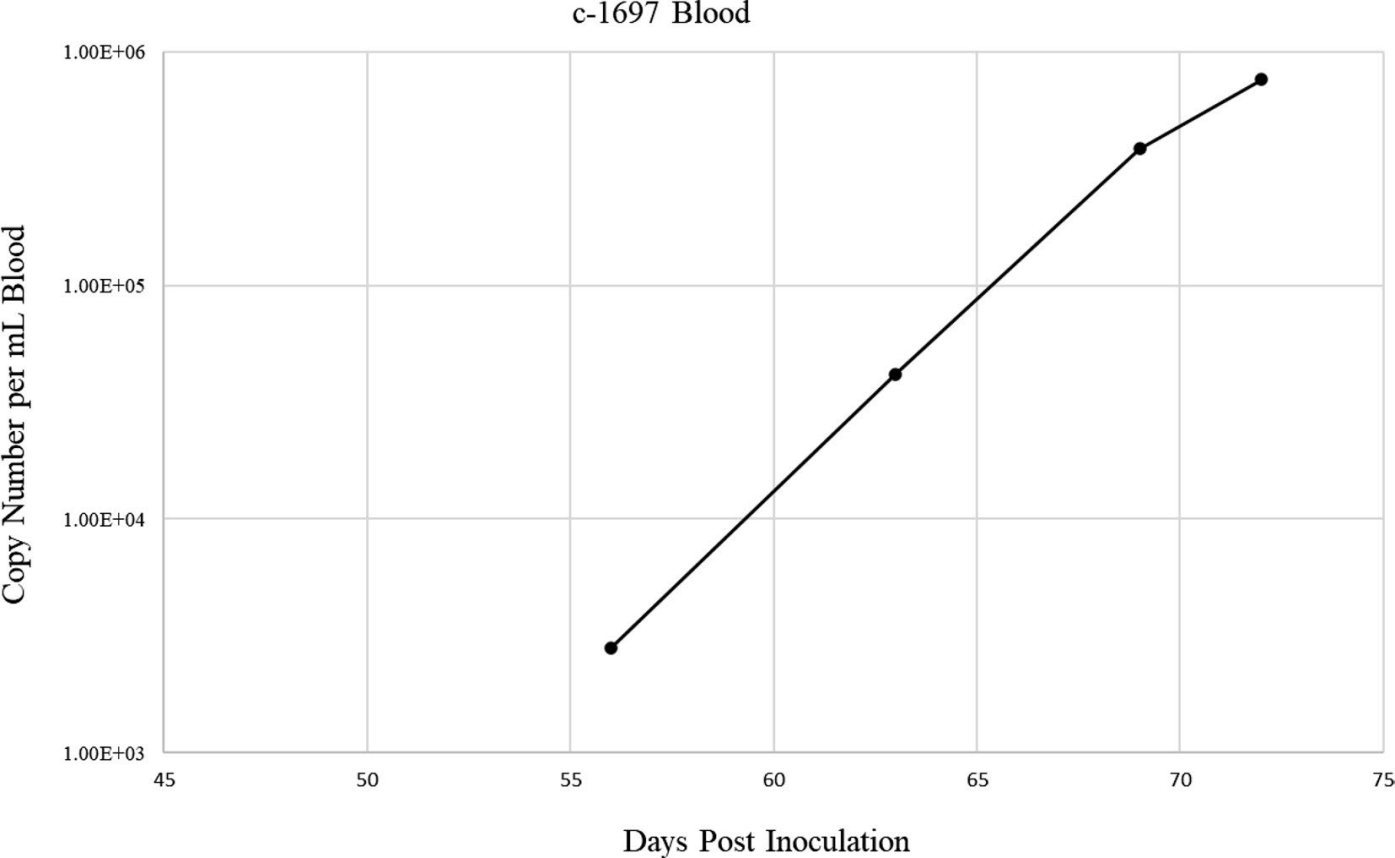
Quantitative PCR of *T. orientalis mpsp *in blood samples from acquisition-fed calf 1697. EDTA-anticoagulated peripheral blood samples were collected during course of infection and evaluated for *T. orientalis* by *mpsp* qPCR. Data points represent the copy number per mL of blood collected on the indicated day post inoculation

Sixty-three days following IV inoculation with *T.* *orientalis* Ikeda blood stabilate, calf 1697 tested positive for *T.* *orientalis* Ikeda via peripheral blood PCR (Additional file 1: Figure S1). *T.* *orientalis* merozoites were detectable in Diff-Quick stained blood smears from day 80 post-infection onward (Fig. 1a). Peripheral blood parasitemia peaked at 3.4% 91 days post infection, then decreased (Fig. 2). The PCV of calf 1697 steadily declined 6%, from 32% pre-infection to a nadir of 26%, by 99 days post-infection (Fig. 2). *T.* *orientalis* was detected by qPCR beginning at 56 days post-infection through the end of the study at 72 days post-infection with detected copy numbers per mL of blood increasing steadily from 2.81 × 10^3^ to 7.61 × 10^5^ (Fig. 3 and Additional file 2:Table S1). As is commonly observed in *T.* *orientalis* infections, the calf exhibited no other clinical signs of piroplasmosis (e.g. fever, anorexia, icterus, and hemoglobinuria) throughout the course of infection.

### *H. longicornis* ticks fed as nymphs on a *T. orientalis* Ikeda-infected calf acquire *T. orientalis* Ikeda infection

Batch #1 nymphs fed to repletion after approximately 4 days on *T.* *orientalis* Ikeda PCR-positive blood (Fig. 2; solid box). Batch #2 nymphs fed to repletion after approximately 8 days, during which peripheral blood parasitemia increased from 0.1 to 1.7% (Fig. 2; dashed box). Of the total nymphs applied, 150/154 from Batch #1 and 114/154 from Batch #2 were collected.

**Fig. 4:**
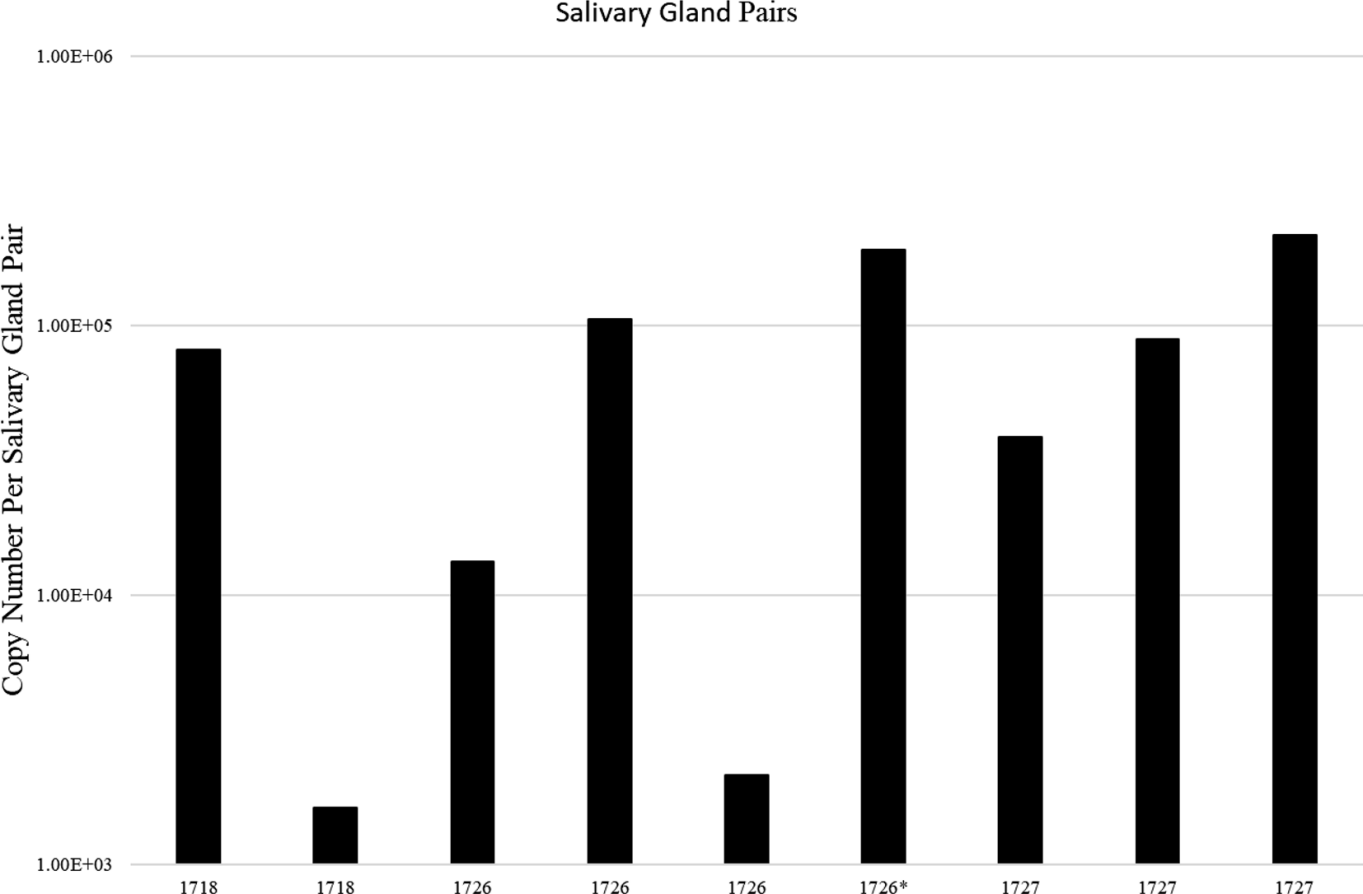
Quantitative PCR of *T. orientalis mpsp* in salivary gland samples from acquisition-fed adult *H. longicornis* ticks. Salivary glands dissected from a subset of adult ticks from Batch #1 and Batch #2 were stimulation fed on calves 1718, 1726, and 1727. Only positive ticks are depicted in the graph. Columns represent the copy number per salivary gland pair dissected from a single tick. *Single positive salivary gland pair from batch #2

After molting, *T.* *orientalis* Ikeda was detected in salivary glands from a subset of adult ticks from both Batch #1 and Batch #2 following a 4 day stimulation feed. Of the subset of stimulation-fed ticks from Batch #1, 8/17 were positive by qPCR with an average infection rate of 47.1 ± 12.4% and a range from 1.63 × 10^3^ to 2.17 × 10^5^ copies per salivary gland pair. From Batch #2, 1/18 was positive representing an infection rate of 5.5% and 1.92 × 10^5^ copies per salivary gland pair. (Fig. 4 and Additional file 2: Table S1).

### *T. orientalis* Ikeda-infected *H. longicornis* adult ticks transmit *T. orientalis* Ikeda to naïve calves

**Fig. 5:**
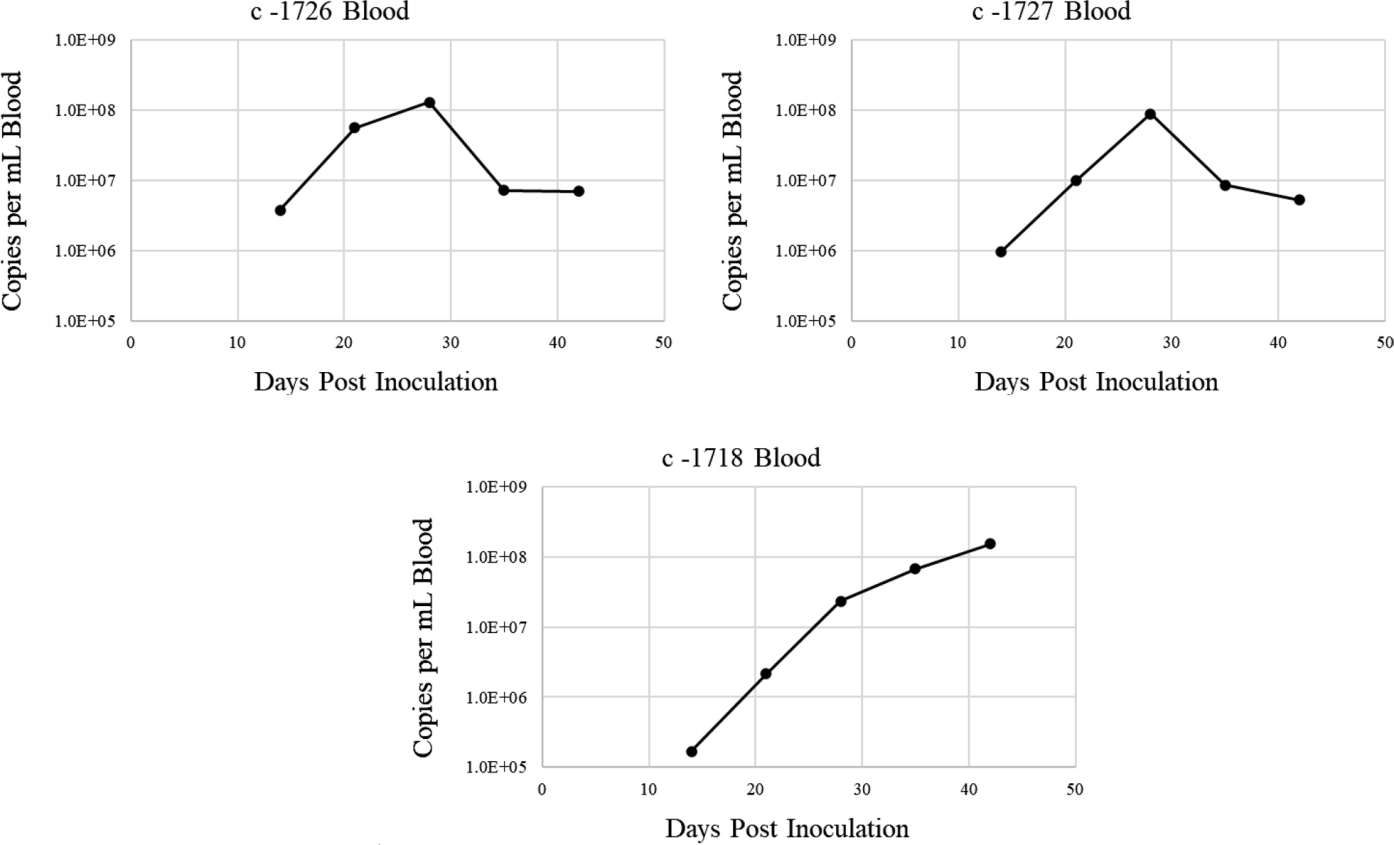
Quantitative PCR analysis of *T. orientalis mpsp* gene fragment in blood samples from transmission-fed calves 1718, 1726, and 1727. Peripheral blood samples were evaluated as described in Fig. 3

**Fig. 6:**
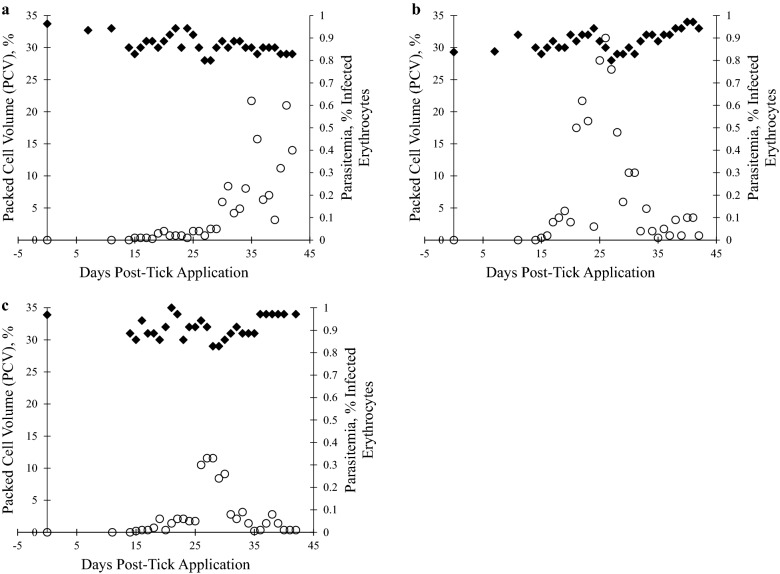
Packed cell volume (PCV) and peripheral blood percent parasitemia of erythrocytes (PPE) in calves 1718, 1726, and 1727 following transmission feed by *T. orientalis* Ikeda-infected *H. longicornis *adult ticks. PCV and Diff-quick-stained blood smears were evaluated following tick attachment and feeding. **a** calf 1718, **b** calf 1726, **c** calf 1727. For A-C, PCV (left y-axis) and PPE (right y-axis) are depicted as percentages, and each value is derived from analysis of a single blood sample taken on the indicated day post-inoculation. ♦ PCV, ○ PPE

Fourteen days after adult tick application, 3/3 spleen-intact calves were positive for *T.* *orientalis* Ikeda via peripheral blood PCR Additional file 1: Figure S1), and merozoites were detected in the erythrocytes of all calves on day 15 post-tick application (Fig. 1b–d). *T.* *orientalis* was detected by qPCR in all three calves from 14 days post-tick application through the end of the study. Copy numbers peaked for two calves (1727 and 1726) at 28 days post-infection while copy numbers were continuing to climb steadily for the third calf (1718) at 42 days post-tick application (Fig. 5), at the end of the study. Copy numbers per mL of blood ranged from 1.68 × 10^5^ to 1.52 × 10^8^ (Additional file 2: Table S1), and for the two calves that demonstrated a peak in detected parasites, the peak was at approximately 1 × 10^8^ per mL of blood (Fig. 5). Peripheral blood parasitemia peaked at 0.4–0.9% between 26 and 31 days post-tick application (Fig. 6), and the PCV of all calves declined 2–7% to nadirs of 28–29%, followed by incremental recovery (Fig. 6). One calf (1727) developed a transient, mild fever (103.5 °F) on day 11 post tick-application, but no other clinical signs of piroplasmosis were exhibited by the calves throughout the course of infection.

### Sequence anzalysis

Consensus sequences for all sequenced amplicons were identical to each other and to that of the Ikeda strain, similar to the analysis by Oakes [23].

## Discussion

These findings confirm that the U.S population of *H.* *longicornis* is a competent vector of *T. orientalis* Ikeda detected in beef cattle in VA, U.S. in 2017 [23]. This is the first report of *T.* *orientalis* transmission by *H.* *longicornis* ticks in the U.S. Our findings support the hypothesis that transmission of *T.* *orientalis* Ikeda within the Abermarle County, VA area is due to the abundance of *H.* *longicornis.* The previous detection of *T.* *orientalis* Ikeda in *H*. *longicornis,* but not native ticks, from the same area in VA provides further support for this hypothesis [33].

Since the *T.* *orientalis* Ikeda outbreak was detected 2017, concomitant with the introduction of *H.* *longicornis* to the U.S., it is possible that the tick and the parasite were imported together (e.g. *T.* *orientalis* Ikeda-infected *H.* *longicornis* ticks were imported); however, it is also possible that they were introduced separately (e.g. *T.* *orientalis*-infected cattle and uninfected *H.* *longicornis* ticks entered separately), and an outbreak occurred when the parasite and the tick arrived in the same area of VA at the same time. In general, although this has yet to be empirically demonstrated in this strain of *T.* *orientalis* Ikeda, *Theileria* sp. are not transovarially transmitted. Furthermore, *H.* *longicornis* survives well in varied environmental conditions [24, 27, 28, 35], and exhibits a wide variety of host feeding preferences, in addition to cattle, including deer and avian species [25, 27, 28, 35]. Thus, if infected ticks were imported, an outbreak of *T.* *orientalis* Ikeda could occur if those infected ticks fed on cattle during their life cycle. In contrast, if the infected ticks completed their life cycle by feeding on other animals, their progeny would likely not be infected with *T.* *orientalis* Ikeda, and no outbreak would occur. This may explain the lack of *T.* *orientalis* Ikeda outbreaks in other areas of the U.S. where *H.* *longicornis* has become established [25, 27, 28, 35].

Given the cosmopolitan nature of the tick vector, there is significant potential for extensive, rapid spread of *T.* *orientalis* Ikeda throughout the U.S. due to movement of infected, asymptomatic cattle. Additionally, other North American tick species competent to transmit *T.* *orientalis* Ikeda are not yet known. Small-scale *T.* *orientalis* Buffeli infections have been detected in cattle herds in different areas of the U.S. since 1955 [1, 21, 22], prior to the introduction of *H.* *longicornis* ticks. It is thus likely some indigenous North American tick species are also capable of transmitting *T.* *orientalis* Ikeda. There is one report of *T.* *orientalis* acquisition and transmission by *Rhipicephalus microplus* ticks in India [12]. As *R.* *microplus* ticks are also native to regions of the U.S. near the southern border, and are competent vectors of *Theileria equi* [36–39]*,* it is possible that they have been involved in *T.* *orientalis* transmission in the past, and could prove competent vectors of *T.* *orientalis* Ikeda. Other North American tick species, including *Amblyomma mixtum, Dermacentor variabilis,* and *Amblyomma americanum*, are competent vectors of other *Theileria* sp. [36, 37, 39, 40], and thus may be competent vectors of *T.* *orientalis* as well. Although *T.* *orientalis* was not detected in field-collected cohorts of *A. americanum *(*n* = 28) and *D. variablilis* (*n* = 10) ticks in VA [33], larger scale, controlled acquisition and transmission experiments are required to definitively determine the vector competence of these tick species for *T.* *orientalis* Ikeda.

In our study, parasitemia was markedly delayed when the calf was infected via IV inoculation with blood stabilate as compared to tick feeding. There are several possible explanations for this observation. First, during tick feeding, infectious parasites are delivered to the bloodstream of the bovine within a milieu of salivary proteins that modulate the immune response and facilitate infection of cells by sporozoites [41–44]. As these modulating proteins are largely absent from infected blood stabilates, infection of host cells by parasites is less efficient. Furthermore, heterologous erythrocyte and lymphocyte antigens in the infected blood stabilate stimulate destruction of the inoculum by the recipient. Second, significant parasite amplification occurs within the tick vector, both via sexual and asexual reproduction [45, 46]. Thus, cattle infected via tick bite likely received a higher dose of *T.* *orientalis* parasites. Third, when tick transmission is used for infection, parasites undergo another round of asexual reproduction within leukocytes of the host (schizogony) [3], further increasing the number of parasites that subsequently infect bovine erythrocytes, bringing animals to the threshold of PCR detection much more quickly. While small numbers of contaminating schizont-infected lymphocytes were likely present in the blood stabilates, their prevalence would have been incomparable to those observed following natural tick transmission, and most of them would have been destroyed by the immune system of the recipient calf due to expression of heterologous lymphocyte antigens. Finally, it is likely that stabilate preparation, shipment, and storage led to parasite degradation, effectively significantly decreasing the infectious parasite dose.

Disease severity due to *T.* *orientalis* Ikeda is variable. The three animals infected via tick feeding in this study developed mild disease in comparison to the cattle from which the isolate was derived, likely due to reduced stress, lack of exposure to other pathogens, and, most importantly, reduced parasite dose. In other virulent *Theileria* species, including *T.* *parva* and *T.* *annulata*, parasite dose is a critical determinant of disease lethality [47, 48]. In *T.* *orientalis*-endemic areas, cattle often endure dense *H.* *longicornis* infestations [49], and thus may be exposed to extremely high numbers of parasites. Exposure of immunologically naïve or debilitated animals to such a high parasite dose may contribute to morbidity and mortality caused by *T.* *orientalis*. Cattle age and pregnancy status are also purported to play a role in *T.* *orientalis* disease susceptibility. For instance, in Australia and New Zealand, the majority of *T.* *orientalis-*related economic impact on the cattle industry is due to fetal loss, abortion, and neonatal calf death [16, 20, 50].

## Conclusions

In conclusion, we have demonstrated that a U.S. population of the invasive Asian longhorned tick, *H.* *longicornis*, is a competent vector of the *T.* *orientalis* Ikeda genotype isolated from a beef herd outbreak in VA, U.S. in 2017 [23]. Since the Asian longhorned tick has become established in fourteen U.S. states [25, 28, 35], and many *T.* *orientalis-*infected cattle exhibit only mild, intermittent clinical signs, there is significant potential for *T.* *orientalis* Ikeda to spread widely within the expanding North American range of *H.* *longicornis* in the absence of surveillance and livestock tick control measures throughout this and neighboring regions. Furthermore, the development and implementation of improved diagnostic assays for *T.* *orientalis* surveillance may be needed to reduce losses incurred by the U.S. cattle industry. Finally, future studies to determine the vector competence of other North American tick species for *T.* *orientalis* Ikeda will provide critical knowledge regarding disease transmission and susceptibility of cattle in different regions of the United States.

## Supplementary Information

**Additional file 1: Figure S1.** Representative agarose gel electrophoresis image of PCR amplified products of the *mpsp* fragment from acquisition- and transmission-fed calf blood samples. Acquisition-fed calf 1697 at 63 dpi and transmission-fed calves 1718, 1726, and 1727 at 14 dpi. POS; *T. orientalis* blood stabilate positive control, NEG; no template control.

**Additional file 2: Table S1.** *T. orientalis* MPSP qPCR raw data files

## Data Availability

All data generated or analyzed during this study are included in this published article (and its additional files).
