# Supplementary figures and images for: A U.S. isolate of Theileria orientalis, Ikeda genotype, is transmitted to cattle by the invasive Asian longhorned tick, Haemaphysalis longicornis
Source: Parasit Vectors. 2021 Mar 16;14:157. doi: 10.1186/s13071-021-04659-9 (PMC7962341; doi:10.1186/s13071-021-04659-9)

## Slide 1
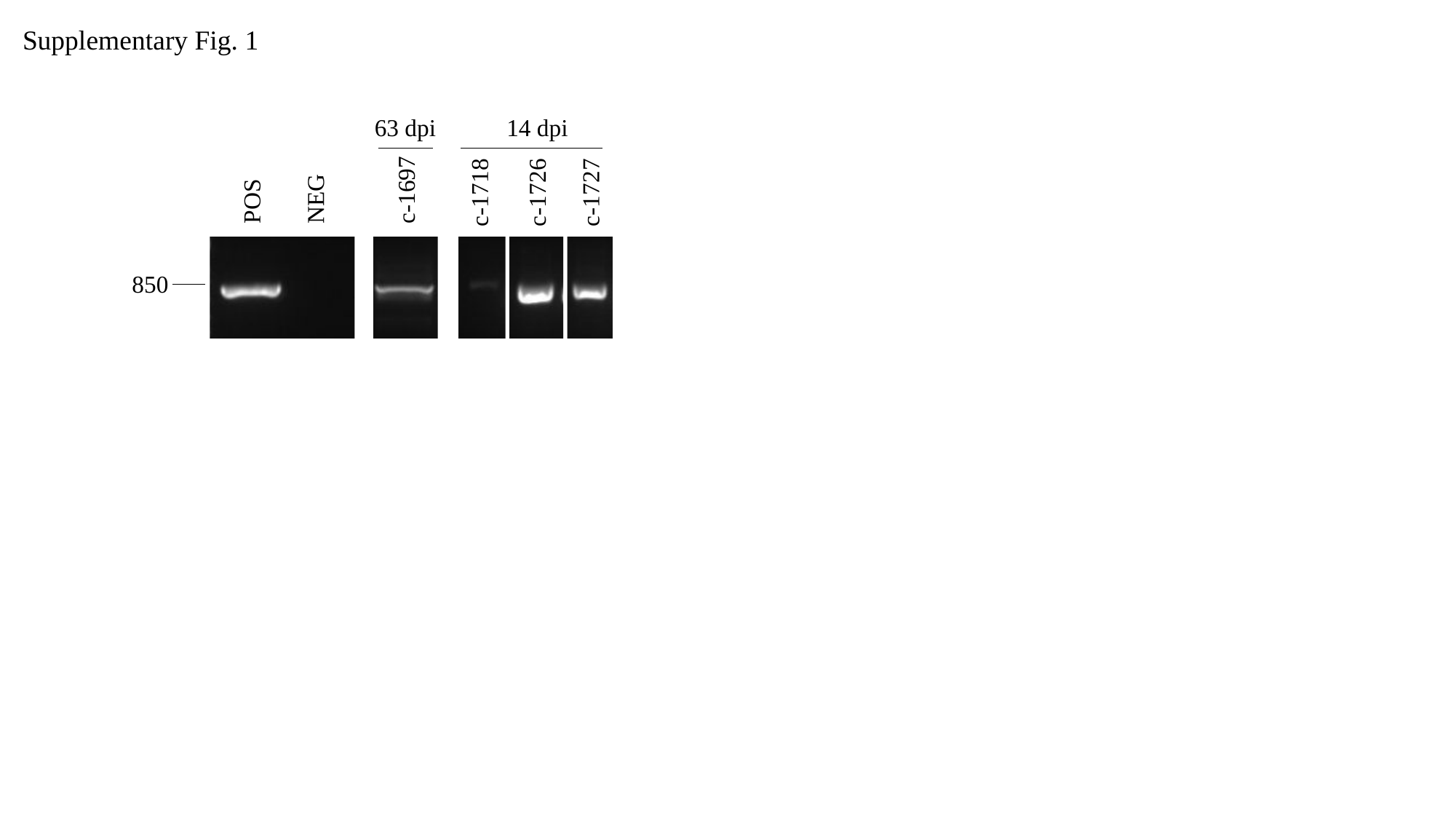

Supplementary Fig. 1
63 dpi
14 dpi
POS
NEG
c-1697
c-1718
c-1726
c-1727
850

Supplement: Supplementary file 1 — Additional file 1: Figure S1. Representative agarose gel electrophoresis image of PCR amplified products of the mpsp fragment from acquisition- and transmission-fed calf blood samples. Acquisition-fed calf 1697 at 63 dpi and transmission-fed calves 1718, 1726, and 1727 at 14 dpi. POS; T. orientalis blood stabilate positive control, NEG; no template control. [file 13071_2021_4659_MOESM1_ESM.pptx]
